# Safety, effectiveness and the optimal duration of preoperative imatinib in locally advanced gastric gastrointestinal stromal tumors: A retrospective cohort study

**DOI:** 10.1002/cam4.70237

**Published:** 2024-09-20

**Authors:** Xiangfei Sun, Xiaohan Lin, Qiang Zhang, Chao Li, Ping Shu, Xiaodong Gao, Kuntang Shen

**Affiliations:** ^1^ Department of General Surgery, Zhongshan Hospital Fudan University School of Medicine Shanghai China; ^2^ Department of General Surgery The First Affiliated Hospital of Nanjing Medical University Nanjing China

**Keywords:** gastrointestinal stromal tumors, imatinib, neoadjuvant treatment, prognosis, stomach

## Abstract

**Background:**

The optimal duration of preoperative imatinib (IM) remains controversial. This study aimed to evaluate the safety, therapeutic effectiveness, and optimal duration of preoperative IM in patients with locally advanced gastric gastrointestinal stromal tumors (GIST).

**Methods:**

The clinicopathologic data of 41 patients with locally advanced gastric GIST who received preoperative IM and underwent surgical resection from January 2014 and December 2021 were retrospectively analyzed.

**Results:**

After a median of 7.0 (IQR: 4.5–10) months of preoperative IM treatment, 30 patients experienced adverse events (AEs), 80% of which were grade 1/2 AEs. The mean tumor size decreased from 12.71 ± 5.34 cm to 8.26 ± 4.00 cm, with a reduction rate of 35%. Setting 8 months as the cut‐off value according to the results of ROC analysis. The proportion of laparoscopic surgery was higher in patients with short‐term (≤8 months) versus long‐term (>8 months) preoperative IM. Compared with the subtotal/total gastrectomy group, patients in the local gastrectomy group exhibited less intraoperative blood loss, shorter length of postoperative hospital stay, and fewer postoperative complications. The 3‐year recurrence‐free survival (RFS) and overall survival (OS) rates were 82.9% and 97.6%, and the expected 5‐year RFS and OS rates were 75.6% and 90.2% respectively. RFS was better in the short‐term than in the long‐term preoperative IM treatment group, and it was also better in pre‐ plus postoperative IM treatment group than that in the preoperative IM alone group. Both univariate and multivariate COX analysis showed that a higher mitotic index and long‐term preoperative IM treatment were associated with worse RFS, while postoperative IM treatment could significantly improve RFS.

**Conclusions:**

The study suggests that in patients with locally advanced gastric GIST, preoperative short‐term (≤8 months) use of IM is associated with higher RFS than long‐term use.

## 1 INTRODUCTION

Gastrointestinal stromal tumors (GIST) are the most common mesenchymal tumors of the digestive tract with a yearly incidence of 10–15 cases per million people.^1^, ^2^, ^3^ The primary tumor occurs in almost any part of the gastrointestinal (GI) tract, most commonly in the stomach (60%–70%), followed by the small intestine (20%–30%), colorectum (5%), esophagus (1%) and other parts of the GI system.^4^ GIST have unique molecular signatures that are driven by continuously activating mutations of the KIT or platelet‐derived growth factor receptor alpha (PDGFRA) gene in 85%–90% of cases.^5^

Complete surgical resection with histologically negative margins (R0) remains the mainstay of treatment for primary GIST at present.^6^, ^7^ However, over 50% of patients with primary GIST who underwent complete resection eventually experienced recurrence or metastasis.^8^ Facing this dilemma, several risk stratification systems have been established, which include tumor size, tumor location, tumor rupture, and mitotic index, among others.^9^, ^10^, ^11^ Since the first use of imatinib (IM), it has been approved for adjuvant treatment after R0 resection of GIST with median‐ or high‐risk patients, and for the treatment of metastatic or unresectable GIST.^12^, ^13^

Although most GIST cases are resectable at the first visit, a great number of patients are either locally advanced or demand complex operations which may lead to postoperative complications or mortality.^8^ Preoperative treatment using IM is advantageous in that it can debulk the tumor, improve the R0 resection rate, increase the organ preservation rate, decrease the extent of resection, avoid multivisceral resection, and reduce local recurrence.^14^, ^15^ Current studies have shown that the duration of maximum responses may be at 6–12 months,^16^, ^17^, ^18^ while Wang et al.^19^ reported that the median time required for achieving the earliest PR was 3.7 months. Still, the optimal duration of preoperative IM remains controversial, and there is no clear evidence to determine the timing of achieving the optimal therapeutic response before surgery. This study aimed to determine the safety, therapeutic effectiveness, and optimal duration of preoperative IM in patients with locally advanced gastric GIST.

## 2 METHODS

### 2.1 Patient selection and preoperative management

Ethical approval for this study was provided by the Clinical Research Ethics Committee of our hospital on January 16, 2020 (Approval No. B2018‐297(2)). This retrospective study was conducted on patients with locally advanced gastric GIST treated with preoperative IM and underwent surgery between January 2014 and December 2021. The reasons for preoperative IM therapy in these patients include (1) patients who were difficult to achieve R0 resection; (2) patients with massive tumors >10 cm or tumors that required multivisceral and extensive resection in whom intraoperative bleeding, rupture, or iatrogenic dispersion were likely to occur; (3) patients whose tumors were located in special anatomical sites such as the gastroesophageal junction so that the function of the organ was easily damaged by surgery; and (4) patients at risk of postoperative recurrence, complications, or mortality. The exclusion criteria were (1) patients with recurrent and metastatic diseases; (2) patients with missing information; (3) nonsurgical patients; and (4) patients with co‐existing malignancies.

### 2.2 Response assessment of the preoperative treatment

Treatment response was assessed using a modification of response criteria in solid tumors (RECIST 1.1).^20^ Progressive disease (PD) was defined as ≥20% increase in tumor size, stable disease (SD) as <20% increase to <30% decrease, partial response (PR) as ≥30% decrease, and complete response (CR) as complete disappearance of the lesion. The number of mitoses was counted by a pathologist from 50 high‐power fields (HPF). All research procedures were performed under the Institutional Review Board‐approved protocol. The work has been reported in line with the STROCSS criteria.^21^

### 2.3 Surgical procedures

According to the classification criteria of EORTCSTBSG,^22^ surgery was divided into local gastrectomy (LG) and subtotal/total gastrectomy (sTG/TG) in terms of the type, and laparotomy and laparoscopy in terms of the method.

### 2.4 Postoperative IM treatment and follow‐up

For adjuvant treatment, IM was started at 400 mg/day for each patient, and the dose remain the same for the entire dosing period until serious adverse events occur. The method of follow‐up has been described in our previous article.^23^ The last follow‐up was performed in June 2023.

### 2.5 Statistical analysis

Continuous variables are described as the mean ± standard deviation (SD) and were analyzed using the Student *t*‐test or Mann–Whitney *U* test. Categorical variables are described as percentages (%) and analyzed using the Chi‐square test, continuity correction, or Fisher's exact tests according to specific conditions. Survival curves were estimated using the Kaplan–Meier method and compared by log‐rank analysis between groups. Univariate and multivariate analyses were conducted by the COX regression model. *p* ≤ 0.05 was considered statistically significant. All statistical analyses were performed with SPSS® version 26.0 (IBM, Armonk, New York, USA) and GraphPad Prism 8.0.2 (GraphPad Prism Software Inc).

## 3 RESULTS

### 3.1 Baseline clinicopathological data of the patients

**FIGURE 1:**
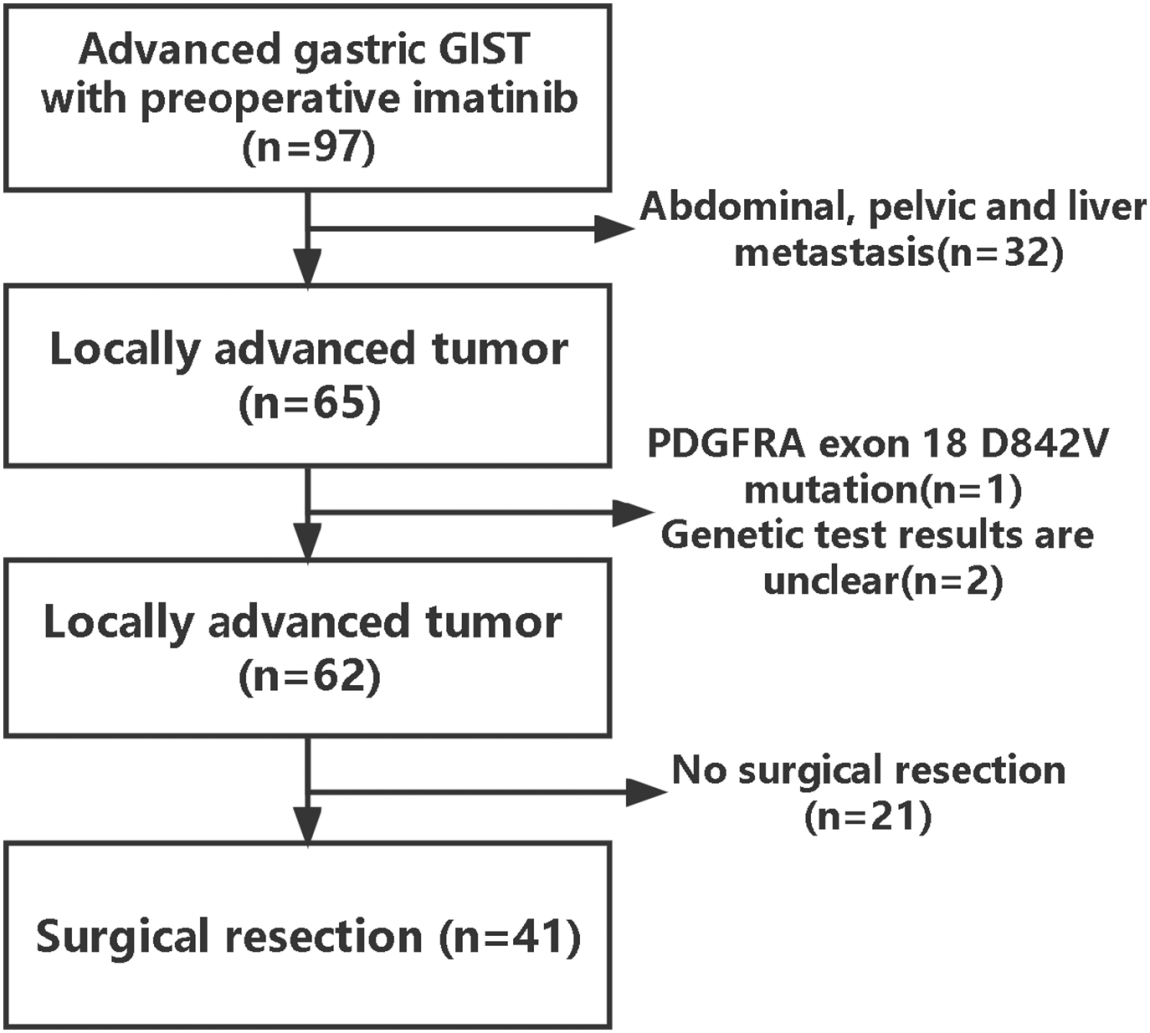
Flow diagram of the cohort of patients with locally advanced gastric gastrointestinal stromal tumors (GIST).

As shown in Figure 1, 97 patients with advanced gastric GIST were diagnosed and treated in our hospital from January 2014 and December 2021. After re‐evaluation by pathologists, all patients were diagnosed with GIST. After excluding 32 patients with abdominal or hepatic metastases at the time of diagnosis, one IM‐insensitive patient with PDGFRA exon 18 D842V mutations, and two patients with indeterminate genetic test results, 62 patients received preoperative IM, of whom 21 patients who were still receiving preoperative IM treatment and had not yet received surgery were excluded from this study according to the exclusion criteria. Finally, 41 patients with locally advanced gastric GIST were included in this study.

The median age of the patients at diagnosis was 61 (IQR, 50–66) years with males predominating (26/41, 63.4%). Only 10 patients (12.7%) were asymptomatic, in whom the tumors were found incidentally. Twenty‐three patients (56.1%) had nonspecific symptoms such as abdominal distension, abdominal pain, nausea, and vomiting. Eight patients (19.5%) had tumor‐specific symptoms including dysphagia, hematemesis, and melena. The mean tumor size at diagnosis was 12.71 ± 5.34 cm. Histologically, 33 cases (80.5%) were classified as the spindle type, three cases (7.3%) as the epithelial type, and five cases (12.2%) as the mixed type. Of the 41 gastric GISTs, 9 (22.0%) originated from the cardia, 7 (17.1%) from the fundus, 20 (48.8%) from the corpus, and 5 (12.2%) from the pylorus. For mutation analysis, we reanalyzed 41 patients subject to sufficient biopsy availability. All patients had KIT gene exon 11 mutations, including point mutation in 19 cases (46.3%), deletion mutation in eight cases (19.5%), insertion mutation in four cases (9.8%), duplication mutation in five cases (12.2%), and mixed mutation in five cases (12.2%). The demographic, clinical, and histopathological features available are listed in Table 1.

**TABLE 1:** Correlation between different preoperative imatinib durations and clinicopathological features in patients with locally advanced gastric GIST.

Factors	Overall (*n* = 41)	Preoperative imatinib		*p*‐Value
		≤8 months (*n* = 27)	>8 months (*n* = 14)	
Gender				
Female	15 (36.6%)	11 (40.7%)	4 (28.6%)	0.443
Male	26 (63.4%)	16 (59.3%)	10 (71.4%)	
Age (years)				
≤60	19 (46.3%)	12 (44.4%)	7 (50%)	0.735
>60	22 (53.7%)	15 (55.6%)	7 (50%)	
Tumor location				
Corpus	20 (48.8%)	13 (48.1%)	7 (50%)	0.910
Noncorpus	21 (51.2%)	14 (51.9%)	7 (50%)	
Tumor size^a^				
≤10 cm	16 (39.0%)	12 (44.4%)	4 (28.6%)	0.323
>10 cm	25 (61.0%)	15 (55.6%)	10 (71.4%)	
Mitotic index^a^				
≤10/50HPF	20 (48.8%)	13 (48.1%)	7 (50%)	0.910
>10/50HPF	21 (51.2%)	14 (51.9%)	7 (50%)	
Morphology				
Spindle	33 (80.5%)	24 (88.9%)	9 (64.3%)	0.142
Epithelioid and Mixed	8 (19.5%)	3 (11.1%)	5 (35.7%)	
KIT exon 11 gene mutation^a^				
Point	19 (46.3%)	17 (63.0%)	2 (14.3%)	*0.003*
Nonpoint	22 (53.7%)	10 (37.0%)	12 (85.7%)	
Surgical method				
Laparotomy	28 (68.3%)	15 (55.6%)	13 (92.9%)	*0.038*
Laparoscopy	13 (31.7%)	12 (44.4%)	1 (7.1%)	
Surgical type				
Local gastrectomy	31 (75.6%)	21 (77.8%)	10 (71.4%)	0.948
Subtotal/Total gastrectomy	10 (24.4%)	6 (22.2%)	4 (28.6%)	

*Note*: All of our variables with *p* values less than 0.05 are in italics.

Abbreviation: HPF, high‐power fields.

^a^ Tumor size, mitotic index, and gene mutations are dependent on data at diagnosis.

### 3.2 Safety of preoperative IM for locally advanced gastric GIST

Forty‐one patients received preoperative IM treatment at a dose of 400 mg per day with a median duration of 7.0 (IQR: 4.5–10) months. All adverse events (AEs) that occurred during preoperative IM treatment are shown in Table 2. A total of 30 patients experienced AEs of various grades, and no patient discontinued the preoperative IM treatment due to AEs. All AEs improved after treatment, and no treatment‐related death occurred. The most common nonhematologic AE was edema (any grade, 73%), followed by skin rash (any grade, 54%). The most common hematologic AE was neutropenia (any grade, 61%), followed by anemia (any grade, 49%). Most AEs were grade 1/2 and the incidence of grade 3 AEs was 15% (6/41), including neutropenia in two cases, nausea, and, vomiting in one case, skin rash in two cases and edema in one case. No grade 4 AE was observed.

**TABLE 2:** Adverse effects during preoperative imatinib treatment in 41 patients with locally advanced gastrointestinal stromal tumor.

Adverse effects	All grade	Grade 1	Grade 2	Grade 3	Grade 4
Abdominal pain	5 (12%)	3 (7%)	2 (5%)	0	0
Nausea/Vomiting	13 (32%)	10 (24%)	2 (5%)	1 (2%)	0
Diarrhea	7 (17%)	5 (12%)	2 (5%)	0	0
Neutropenia	25 (61%)	18 (44%)	5 (12%)	2 (5%)	0
Thrombocytopenia	3 (7%)	3 (7%)	0	0	0
Anemia	20 (49%)	18 (44%)	2 (5%)	0	0
Impaired liver function	18 (44%)	15 (37%)	3 (7%)	0	0
Fatigue	15 (37%)	10 (24%)	5 (12%)	0	0
Skin rash	22 (54%)	17 (41%)	3 (7%)	2 (5%)	0
Oedema	30 (73%)	23 (56%)	6 (15%)	1 (2%)	0
Alopecia	4 (10%)	2 (5%)	2 (5%)	0	0

*Note*: Adverse events of imatinib therapy were assessed according to the National Cancer Institute Common Terminology Criteria for Adverse Events (NCI‐CTCAE V5.0).

### 3.3 Effectiveness of preoperative IM treatment for locally advanced gastric GIST

**FIGURE 2:**
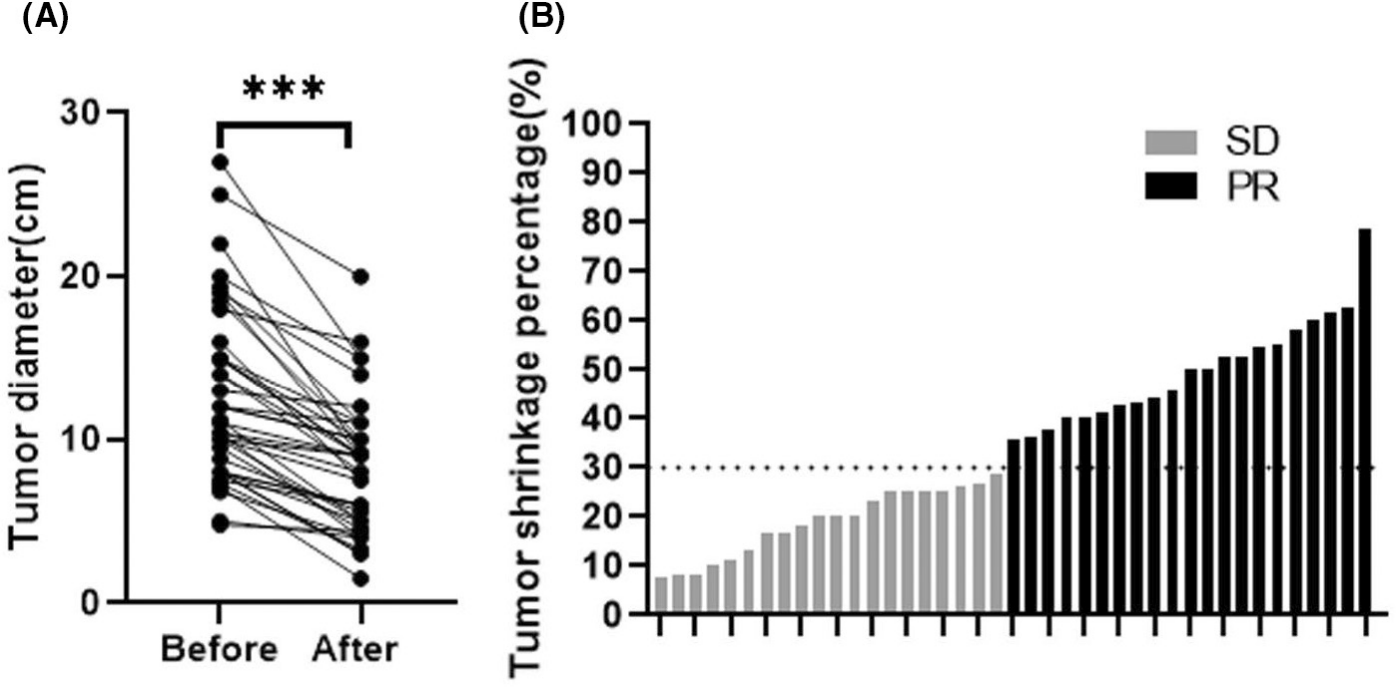
The effectiveness of preoperative imatinib treatment for locally advanced gastric gastrointestinal stromal tumors (GIST). (A) The maximum diameter of locally advanced gastric GIST before and after preoperative imatinib. (B) Waterfall plot of the tumor shrinkage percentage after preoperative imatinib. Stable disease (SD) and partial response (PR) were defined as <20% increase to <30% decrease and ≥ 30% decrease (RECIST 1.1). ***p < 0.001.

**FIGURE 3:**
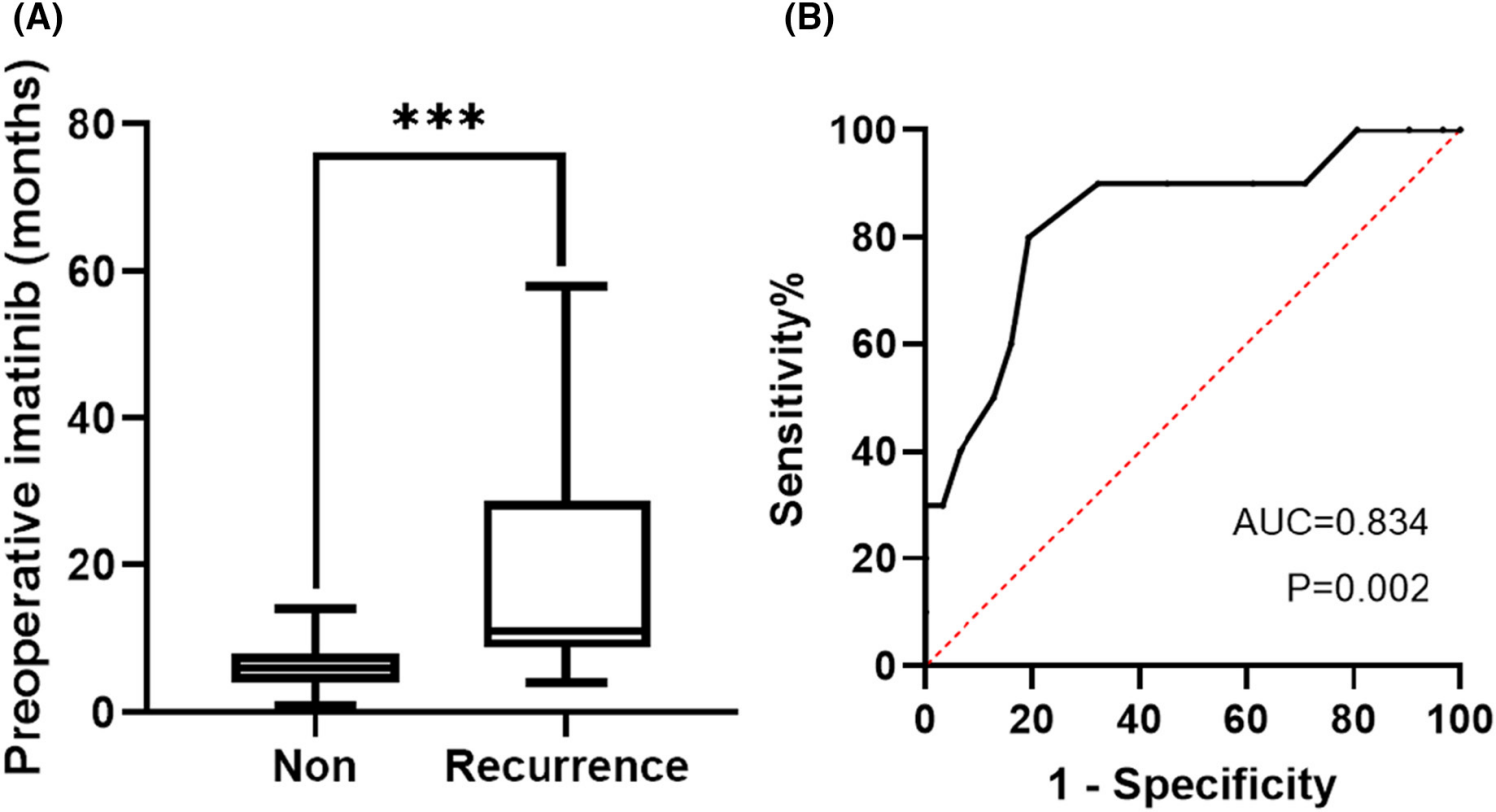
Correlation between preoperative imatinib and tumor recurrence in 41 patients with locally advanced gastric gastrointestinal stromal tumors (GIST). (A) Patients with tumor recurrence had a longer duration of preoperative IM treatment than those without recurrence; (B) ROC curve of preoperative imatinib times predicting recurrence‐free survival in locally advanced gastric GIST patients. ***p < 0.001.

**FIGURE 4:**
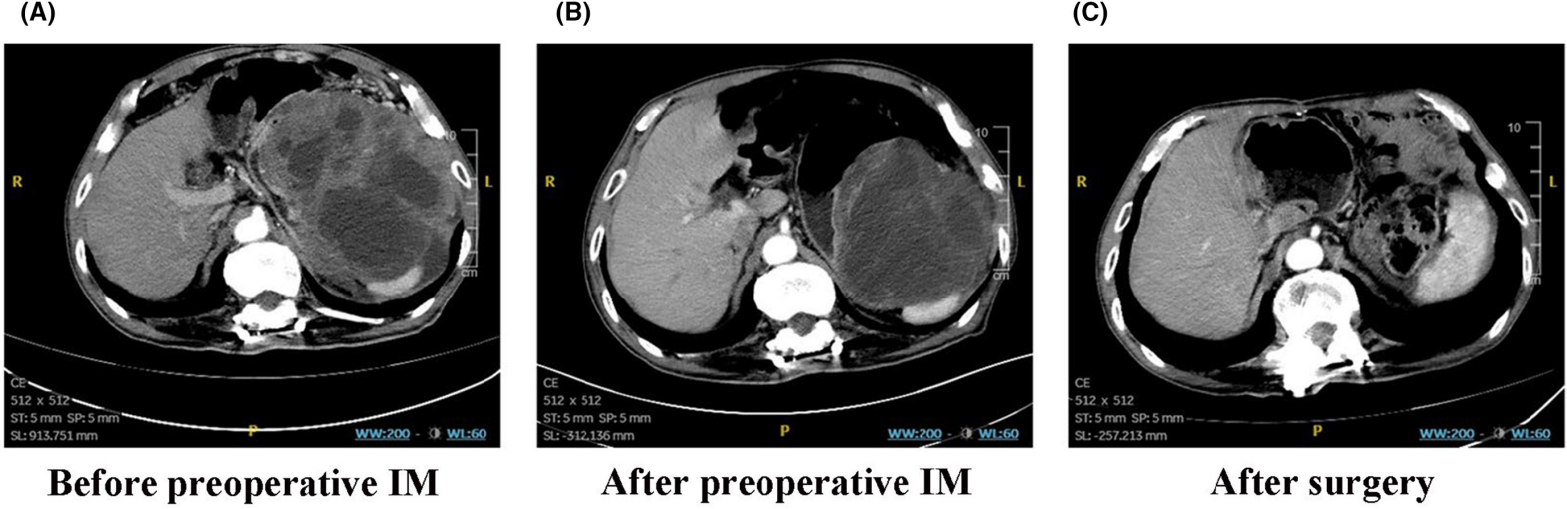
Contrast‐enhanced abdominal CT imaging in a 73‐year‐old male patient with locally advanced gastric gastrointestinal stromal tumors who received 8 months of preoperative imatinib. (A) Before preoperative imatinib treatment (maximum tumor diameter: 19 cm); (B) After 8 months of preoperative imatinib treatment (maximum tumor diameter: 14 cm); (C) 3 months after local gastrectomy.

Preoperative IM was temporarily discontinued for a median of 13 days (range: 7–16 days) before surgery. According to RECIST1.1 criteria,^22^ 21 patients (51.2%) achieved PR, and 20 patients (48.8%) showed SD. The mean tumor size of these patients was significantly reduced from 12.71 ± 5.34 cm to 8.26 ± 4.00 cm (*p* < 0.001) (Figure 2). The median values for tumor size before and after preoperative IM treatment were 11.2 (4.8–27) cm and 9 (1.5–20) cm. We found a highly significant correlation between preoperative IM and tumor recurrence (Figure 3A). While patients without recurrence had a median duration of preoperative IM treatment of 6 months versus 19 months in those with recurrence (*p* < 0.001). We further performed a Receiver Operating Characteristic (ROC) analysis to determine the optimal cut‐off point for the duration of preoperative IM treatment. The ROC curve helps in identifying the threshold that best distinguishes between patients with better and worse RFS (Figure 3B). The ROC analysis identified 8 months as the optimal cut‐off point (sensitivity, 0.800; specificity, 0.806; area under the curve, 0.834), with the highest Youden Index (sensitivity + specificity − 1, 0.606). This statistical measure indicates the point where the balance between sensitivity and specificity is maximized, providing the most accurate division between short‐term and long‐term treatment groups. Figure 4 shows abdominal contrast‐enhanced CT imaging of a 73‐year‐old man with locally advanced gastric GIST. After an 8‐month preoperative IM treatment, the tumor size decreased from 19 cm to 14 cm, and finally LG was performed.

### 3.4 Resection of locally advanced gastric GIST after IM Treatment

Among the 41 patients with locally advanced gastric GIST, 31 patients received LG and 10 patients received sTG/TG. The rate of organ preservation was 78.0%. No intraoperative tumor bleeding or rupture occurred. For patients who failed to preserve adjacent organs, additional operations included splenectomy alone (*n* = 2), distal pancreatectomy and splenectomy (*n* = 4), partial diaphragmectomy (*n* = 1) and lower esophagectomy (*n* = 2). The intraoperative blood loss in LG group was lower than that in sTG/TG group (137.1 ± 33.9 vs. 335.0 ± 417.0, *p* = 0.043). The mean length of postoperative hospital stay was 10.00 ± 4.67 days in all patients (7.23 ± 2.42 days in LG group and 13.8 ± 8.86 days in SR group, *p* = 0.018). In addition, postoperative complications were classified using the Clavien‐Dindo classification.^24^ In LG group, five patients (16.1%) developed postoperative complications, including delayed gastric emptying in three patients, which was cured after conservative treatment, and wound infection in the other two patients, which was cured by local drainage. In sTG/TG group, six patients (60.0%) developed postoperative complications, including high fever due to local infection in three patients, which was cured after antibiotic treatment. Of the four patients who underwent pancreatectomy, three developed pancreatic fistula. Compared with LG group, sTG/TG group had more postoperative complications (*p* = 0.040). No death occurred in the early postoperative period.

### 3.5 Relationship between preoperative IM treatment time and clinicopathological features

We analyzed the correlation between preoperative IM treatment time and clinicopathological features in the 41 patients with locally advanced gastric GIST. Using the treatment time of 8 months as the cut‐off according to the results of ROC analysis, the patients were divided into two groups: a short‐term preoperative treatment group and a long‐term preoperative treatment group. The basic clinicopathological characteristics of the two groups are shown in Table 1. The results showed that the time of preoperative IM treatment was correlated with tumor gene mutation (*p* = 0.003) and surgical methods (*p* = 0.038). The proportion of laparoscopic surgery was higher in patients with less than or equal to 8 months of preoperative treatment (44.4% vs. 7.1%, *p* = 0.038).

### 3.6 Postoperative IM treatment

Of the 41 patients receiving preoperative IM treatment, 5 (12.2%) received preoperative IM only, and 36 (87.8%) received postoperative IM treatment for a median duration of 35 (IQR, 26.8–48.3) months at a dose of 400 mg per day. Out of these five patients who did not receive postoperative IM, two were in the short‐term IM group and three were in the long‐term IM group. We have conducted a further analysis to check for any imbalances between the two groups regarding postoperative IM administration. The results showed that while there was a slight numerical difference, statistical tests indicated no significant imbalance between the groups (*p* > 0.05). The clinical outcome in terms of RFS in the preoperative treatment combined with the postoperative IM treatment group was better than that in the preoperative treatment alone group (Log‐rank, *p* < 0.001) (Figure 6C). However, postoperative IM had no significant impact on OS (Log‐rank, *p* = 0.150) (Figure 6D).

### 3.7 Follow up

**FIGURE 5:**
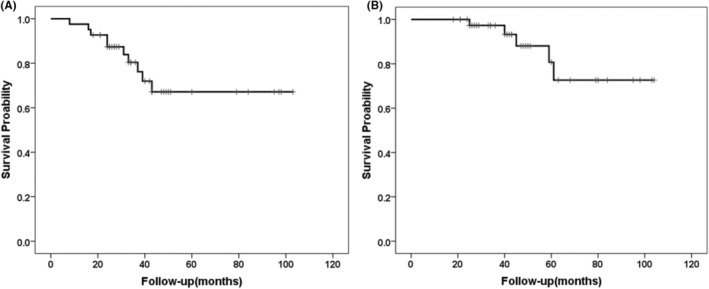
Recurrence‐free survival (A) and overall survival (B) curves of 41 patients with locally advanced gastric gastrointestinal stromal tumors.

**FIGURE 6:**
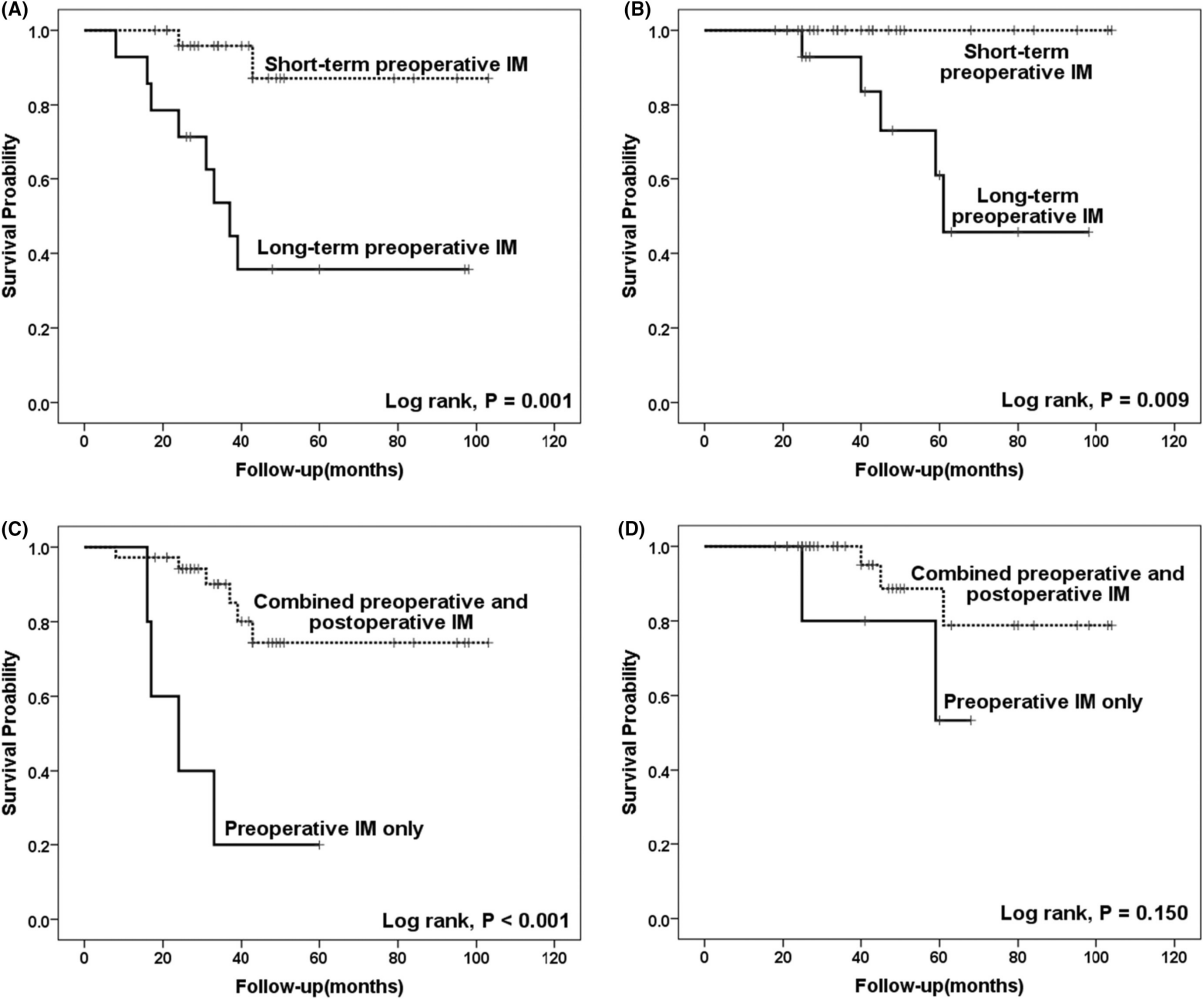
Kaplan–Meier analysis of the relationships between preoperative and postoperative imatinib (IM) treatment and recurrence‐free survival (RFS) and overall survival (OS). (A) Locally advanced gastric gastrointestinal stromal tumors (GIST) patients receiving short‐term (≤8 months) preoperative IM had a better RFS (A) and OS (B) than those receiving long‐term(>8 months) preoperative IM treatment; (C) RFS was significantly longer in patients with postoperative IM than that in patients without postoperative IM treatment; (D) Postoperative IM had no significant effect on OS.

**FIGURE 7:**
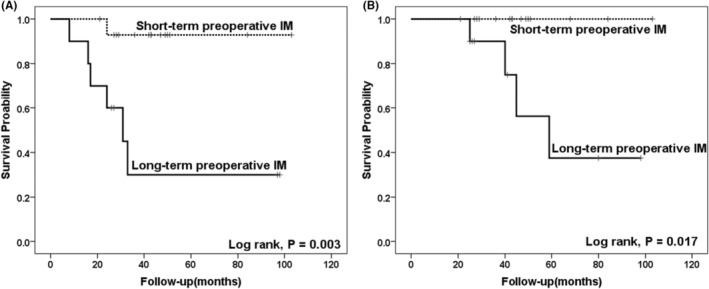
Kaplan–Meier analysis of the relationships between preoperative imatinib (IM) treatment and recurrence‐free survival (RFS) and overall survival (OS) in subgroups of tumors greater than 10 cm. Compared with long‐term preoperative IM treatment, short‐term IM treatment further improved the RFS (A) and OS (B) of GIST patients with tumors larger than 10 cm.

By June 2023, all 41 patients had been followed up for a median of 42 (IQR, 27.0–60.5) months, during which relapse occurred in 10 cases (24.4%) and disease‐related death occurred in five cases (12.2%). The most common site of metastasis was the liver (*n* = 7), followed by the retroperitoneum (*n* = 2) and esophagus (*n* = 1). The 3‐year RFS rate and OS rate were 82.9% and 97.6%, respectively; the expected 5‐year RFS rate and OS rate were 75.6% and 90.2% (Figure 5). In the short‐term preoperative treatment group (less than or equal to 8 months), there were 2 recurrences and no death cases. In the long‐term preoperative treatment group (more than 8 months), eight cases relapsed, and five cases of death due to disease progression. The duration of preoperative IM treatment had a significant impact on RFS and OS, and the clinical outcome in the long‐term preoperative treatment group was worse in terms of RFS (Log‐rank, *p* = 0.001) (Figure 6A) and OS (Log‐rank, *p* = 0.009) (Figure 6B) compared with the short‐term preoperative IM treatment group. Analysis of the subgroup of tumors greater than 10 cm showed that compared with long‐term preoperative IM treatment, short‐term IM treatment further improved the RFS (Log‐rank, *p* = 0.003) and OS (Log‐rank, *p* = 0.017) of GIST patients (Figure 7).

Univariate and multivariate Cox regression models were used to determine the impact of RFS and OS factors. Univariate Cox analysis showed that RFS was associated with tumor mitotic index (HR: 5.559, 95% CI: 1.170–26.148, *p* = 0.031), preoperative IM treatment time (HR: 9.056, 95% CI: 1.921–42.702, *p* = 0.005), surgical type (HR: 5.270, 95% CI: 1.472–18.865, *p* = 0.011) and postoperative IM (HR: 0.137, 95% CI: 0.038–0.492, *p* = 0.002) (Table 3). To minimize the impact of selection bias on RFS, multivariate Cox regression analysis was performed, and the results showed that mitotic figures, preoperative IM treatment time and postoperative IM treatment were three independent factors affecting RFS in patients with locally advanced gastric GIST. A higher mitotic index (HR: 47.727, 95% CI: 2.484–916.864, *p* = 0.010) and a longer duration of preoperative IM treatment (HR: 42.924, 95% CI: 2.053–897.340, *p* = 0.015) were associated with worse RFS, while postoperative IM treatment (HR: 0.042, 95% CI: 0.004–0.449, *p* = 0.009) could significantly improve RFS of the patients (Table 3). In addition, univariate Cox regression analyses showed that there was no correlation between OS and various factors (Table S1).

**TABLE 3:** Univariate and multivariate Cox regression analyses on variables affecting recurrence‐free survival of patients with locally advanced gastrointestinal stromal tumors.

Factors	Univariate		Multivariate	
	HR (95% CI)	*p* Value	HR (95% CI)	*p* Value
Gender				
Female	1	0.736	—	—
Male	0.792 (0.204–3.070)			
Age (years)				
≤60	1	0.391	—	—
>60	1.745 (0.489–6.228)			
Location				
Corpus	1	0.706	—	—
Noncorpus	1.276 (0.360–4.525)			
Tumor size				
≤10 cm	1	0.505	1	0.245
>10 cm	1.586 (0.409–6.148)		3.341 (0.438–25.487)	
Mitotic index				
≤10/50HPF	1	*0.031*	1	*0.010*
>10/50HPF	5.559 (1.170–26.148)		47.727 (2.484–916.864)	
Morphology				
Spindle	1	0.089	1	0.554
Epithelioid and Mixed	3.027 (0.845–10.837)		0.564 (0.085–3.747)	
Mutation type of KIT exon11				
Point	1	0.053	1	0.765
Nonpoint	4.644 (0.980–22.004)		0.676 (0.052–8.819)	
Surgical method				
Laparotomy	1	0.699	—	—
Laparoscopy	0.765 (0.196–2.982)			
Surgical type				
Local gastrectomy	1	*0.011*	1	0.320
Subtotal/Total gastrectomy	5.270 (1.472–18.865)		3.215 (0.321–32.159)	
Preoperative imatinib duration				
≤8 months	1	*0.005*	1	*0.015*
>8 months	9.056 (1.921–42.702)		42.924 (2.053–897.340)	
Postoperative imatinib				
No	1	*0.002*	1	*0.009*
Yes	0.137 (0.038–0.492)		0.042 (0.004–0.449)	

*Note*: All of our variables with *p* values less than 0.05 are in italics.

Abbreviation: HPF, high‐power fields.

## 4 DISCUSSION

The emergence of IM has significantly improved the prognosis of patients with advanced GIST, and preoperative IM is supposed to be beneficial for patients with locally advanced primary GIST by shrinking tumors and possibly facilitating R0 resection and organ‐sparing surgery.^25^ In addition, since the primary tumor is rich in blood supply, preoperative IM treatment can reduce the risk of intraoperative tumor rupture, bleeding, and postoperative complications.^26^ Although some published studies^16^, ^18^, ^27^ advocate the benefit of neoadjuvant IM treatment for locally advanced GIST, most of these studies were small clinical trials and included GIST originating from multiple organs such as the small intestine and colorectum. Unlike these studies, our study focused on locally advanced GIST of gastric origin and the result demonstrated that preoperative IM treatment offered good outcomes for this type of GIST. Before preoperative IM treatment, it is not only necessary to clarify the pathological diagnosis, but also strive to clarify the type of gene mutation to determine the dose of IM according to the genotype. As all patients in our series had KIT gene exon 11 mutation, IM treatment at 400 mg per day was prescribed for each patient.

With respect to the safety of preoperative IM treatment, several small prospective series and retrospective studies reported that preoperative IM was associated with higher complication rates in advanced disease.^28^, ^29^, ^30^ However, the results of our study showed that patients generally tolerated preoperative IM treatment well. After a median of 7.0 (IQR: 4.5–10) months of preoperative IM treatment, 30 of the 41 patients experienced AEs of different grades, but 80% of these AEs belonged to grade 1/2, with no grade 4 AEs observed. All AEs were relieved after symptomatic treatment. Therefore, for locally advanced gastric GIST, although the preoperative IM may increase the risk of related complications, careful regular monitoring and timely intervention of AEs by experienced professionals will help ensure patient safety.

RTOG0132/ACRIN6665 is a prospective phase II study evaluating the safety and efficacy of preoperative IM in 30 patients with primary GIST, of whom 7% patients achieved PR and 83% achieved SD. In addition, no significant preoperative IM treatment‐associated complication was reported in their study.^17^ Fiore et al.^31^ reported 15 cases of primary locally advanced GIST, in which the median duration of preoperative IM treatment was 9 months and all patients achieved definite tumor remission, with a median tumor shrinkage rate of 34%. Shen et al.^32^ reported tumor shrinkage in all their 18 patients with primary unresectable GIST after a median 7‐month preoperative IM treatment, with a median tumor size shrinkage rate of 35%. Similar to these findings, the mean tumor size in our 41 patients with locally advanced gastric GIST decreased from 12.71 ± 5.34 cm to 8.26 ± 4.00 cm after preoperative treatment (*p* < 0.001), with a mean shrinkage rate of 35%. Of them, 21 patients achieved PR and 20 patients achieved SD. The proportion of laparoscopic surgery in patients with short‐term preoperative treatment was higher than that in those with long‐term preoperative treatment (*p* = 0.038). it can be concluded that preoperative use of IM is effective and can significantly reduce tumor size, possibly improve the R0 resection rate, and increase the organ preservation rate.

Current studies have shown that the duration of maximal remission with IM preoperative treatment is 6–12 months,^33^, ^34^ Yoon et al.^35^ reported that the maximum benefit time of preoperative IM treatment was 9 months in patients with locally advanced rectal GIST. One study showed that when CT showed no significant change in tumor size or density, the median time to preoperative treatment was 11.9 (2.8–15.0) months.^36^ Most experts tend to perform surgical resection when the tumor has the greatest response to IM treatment (two adjacent consecutive imaging examinations do not show significant changes in the tumor).^37^, ^38^, ^39^ However, long‐term preoperative IM may cause the risk of drug‐resistant tumor progression. So far, there is still no consensus on the optimal duration of preoperative IM treatment for locally advanced gastric GIST. It was found in our study that there was a significant difference in the duration of preoperative IM treatment between recurrent patients and nonrecurrent patients, and the preoperative treatment time of recurrent patients was significantly longer than that in patients without recurrence. ROC analysis showed that the optimal duration of preoperative IM treatment for locally advanced gastric GIST was 8 months (AUC = 0.834, *p* = 0.002).

When maximal remission is achieved with preoperative IM treatment, surgical intervention should be performed promptly. The surgical methods that can be used include LG and sTG/TG. The analytic results in this study showed that compared with the sTG/TG group (*n* = 10), LG group (*n* = 31) exhibited less intraoperative blood loss (*p* = 0.043), shorter length of postoperative hospital stay (*p* = 0.018) and fewer postoperative complications (*p* = 0.040). These findings indicate that LG after preoperative IM treatment is superior to sTG/TG for patients with locally advanced gastric GIST.

For primary resectable GIST, postoperative IM treatment is determined according to the pathology of the resected tumor. However, postoperative tumor size and mitotic index were changed in patients who had received preoperative IM treatment. It is generally believed that for patients with R0 resection, the time of postoperative IM treatment should be determined according to the recurrence risk classification before preoperative IM.^17^, ^40^ In our study, the log‐rank analysis results showed that the clinical outcome was better in terms of RFS in the preoperative treatment combined with postoperative IM treatment group than in the preoperative treatment alone group. The decision not to administer postoperative IM was based on patient‐specific factors such as adverse reactions to the drug or patient's preference, and these factors did not systematically differ between the short‐term and long‐term IM groups. Although the results of RTOG‐S0132/ACRIN6665 and GAP study showed that preoperative IM treatment was safe and effective, there is still a lack of convincing evidence to decide whether it can improve RFS and OS due to the short follow‐up duration.^41^ In our study, the 3‐year RFS rate and OS rate were 82.9% and 97.6%, and the expected 5‐year RFS rate and OS rate were 75.6% and 90.2% respectively. In addition, the clinical outcome in the long‐term preoperative treatment group was worse in terms of RFS and OS compared with the short‐term preoperative IM treatment group. To further verify the prognostic factors, we conducted COX univariate and multivariate analysis, the results showed that higher mitotic index and long‐term preoperative IM treatment were associated with worse RFS, while postoperative IM treatment could significantly improve RFS of the patients. Although postoperative IM did not show a statistically significant impact on OS in our cohort, the observed trend suggests that larger studies are warranted to fully assess this potential benefit.

This study has certain limitations. First, the sample size of this study is not large enough because there were very few patients with locally advanced gastric GIST who used preoperative IM and eventually underwent surgical resection. Next, there are multiple factors, such as tumor size, mitotic index, and tumor location, that may lead to varying durations of preoperative IM use in patients with locally advanced gastric GIST. Therefore, we sometimes need to provide personalized treatment strategies based on the characteristics of the patient and the tumor. Finally, this is a single‐center retrospective study, and the results need to be further verified by prospective multicenter randomized controlled trials.

## 5 CONCLUSION

Preoperative IM treatment for locally advanced GIST is generally safe and effective in that it can achieve effective tumor shrinkage, increase the proportion of laparoscopic surgery, preserve important organ functions, and reduce surgical complications. The study suggests that in patients with locally advanced gastric GIST, preoperative short‐term (≤8 months) use of IM is associated with higher RFS than long‐term use.

## AUTHOR CONTRIBUTIONS

**Xiangfei Sun:** Conceptualization (lead); writing – original draft (lead). **Xiaohan Lin:** Data curation (equal). **Qiang Zhang:** Data curation (equal). **Chao Li:** Data curation (equal). **Ping Shu:** Data curation (equal). **Xiaodong Gao:** Project administration (equal). **Kuntang Shen:** Project administration (equal).

## FUNDING INFORMATION

This study was supported by the National Natural Science Foundation of China (81773080).

## CONFLICT OF INTEREST STATEMENT

The authors declare that there is no conflict of interest in the current study.

## INFORMED CONSENT

Informed consent was acquired from all patients for the acquisition of clinical and pathological information and the use of surgical specimens.

## Supporting information


Table S1.

## Data Availability

The datasets used and analyzed during the current study are available from the corresponding author at reasonable request.
