# Supplementary material for: Safety, effectiveness and the optimal duration of preoperative imatinib in locally advanced gastric gastrointestinal stromal tumors: A retrospective cohort study
Source: Cancer Med. 2024 Sep 20;13(18):e70237. doi: 10.1002/cam4.70237 (PMC11413410; doi:10.1002/cam4.70237)
Supplement: Supplementary file 1 — Table S1. [file CAM4-13-e70237-s001.docx]

**Supplemental Table 1. Univariate and multivariate Cox regression analysis on variables affecting the overall survival of patients with locally advanced gastrointestinal stromal tumors**

| Factors | Univariate | | Multivariate | |
| --- | --- | --- | --- | --- |
|  | HR (95% CI) | P value | HR (95% CI) | P value |
| Gender  Female  Male | 1  0.389（0.043-3.536） | 0.402 | - | - |
| Age(years)  ≤60  >60 | 1  0.652（0.107-3.969） | 0.643 | - | - |
| Location  Corpus  Non-corpus | 1  0.433（0.072-2.622） | 0.362 | - | - |
| Tumor size  ≤10cm  >10cm | 1  2.890（0.318-26.267） | 0.346 | - | - |
| Mitotic index  ≤10/50HPF  >10/50HPF | 1  1.699（0.283-10.207） | 0.562 | - | - |
| Morphology  Spindle  Epithelioid and Mixed | 1  4.625（0.760-28.139） | 0.096 | - | - |
| Mutation type of KIT exon11  Point  Non-point | 1  2.957（0.324-26.994） | 0.337 | - | - |
| Surgical method  Laparotomy  Laparoscopy | 1  0.521（0.058-4.711） | 0.561 | - | - |
| Surgical type  Local gastrectomy  Subtotal/Total gastrectomy | 1  5.919（0.979-35.793） | 0.053 | - | - |
| Preoperative imatinib duration  ≤ 8 months  > 8 months | 1  92.606（0.060->10000） | 0.227 | - | - |
| Postoperative imatinib  No  Yes | 1  0.285（0.046-1.751） | 0.175 | - | - |

All of our variables with p values less than 0.05 are in italics. HPF: high-power fields.
